# Behavioral Consequences and Cortical Reorganization in Homonymous Hemianopia

**DOI:** 10.3389/fnsys.2016.00057

**Published:** 2016-06-28

**Authors:** Sylvie Chokron, Céline Perez, Carole Peyrin

**Affiliations:** ^1^Unité Fonctionnelle Vision and Cognition, Fondation Ophtalmologique RothschildParis, France; ^2^UMR 8242, Laboratoire de Psychologie de la Perception, CNRS and Université Paris-DescartesParis, France; ^3^UMR 5105, CNRS, Laboratoire de Psychologie et NeuroCognition, Université Grenoble AlpesGrenoble, France

**Keywords:** homonymous hemianopia, blindsight, sightblindness, hallucinations, occipital lobe

## Abstract

The most common visual defect to follow a lesion of the retrochiasmal pathways is *homonymous hemianopia* (HH), whereby, in each eye, patients are blind to the contralesional visual field. From a behavioral perspective, in addition to exhibiting a severe deficit in their contralesional visual field, hemianopic patients can also present implicit residual capacities, now usually referred to collectively as *blindsight*. It was recently demonstrated that HH patients can also suffer from a subtle deficit in their ipsilesional visual field, called *sightblindness* (the reverse case of blindsight). Furthermore, the nature of the visual deficit in the contralesional and ipsilesional visual fields, as well as the pattern of functional reorganization in the occipital lobe of HH patients after stroke, all appear to depend on the lesion side. In addition to their contralesional and ipsilesional visual deficits, and to their residual capacities, HH patients can also experience visual hallucinations in their blind field, the physiopathological mechanisms of which remain poorly understood. Herein we review blindsight in terms of its better-known aspects as well as its less-studied clinical signs such as sightblindness, hemispheric specialization and visual hallucinations. We also discuss the implications of recent experimental findings for rehabilitation of visual field defects in hemianopic patients.

## Homonymous Hemianopia: Signs and Symptoms, Etiology and Neuro-Anatomical Correlations

**FIGURE 1:**
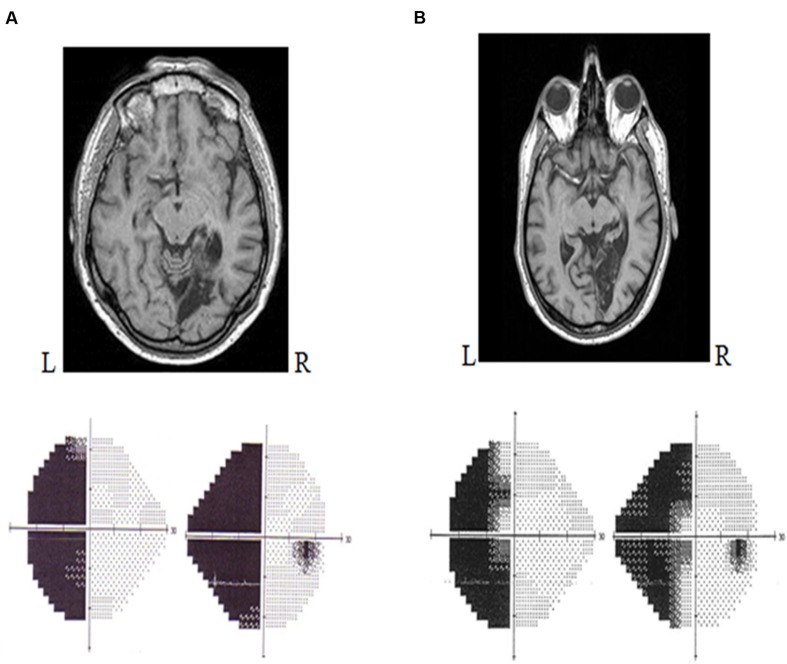
**Humphrey automated perimetry (SITA FAST 24/2).** **(A)** Left homonymous hemianopia without macular sparing. **(B)** Left homonymous hemianopia with macular sparing.

Lesions that occur between the optic chiasm and the primary visual cortex (V1) can provoke a type of visual deficit known as a *cortical visual impairment*. Such deficits translate to visual field defects or to more complicated visual defects, depending on the location of the lesion. Clinically, the most common visual field defect to follow a retrochiasmatic lesion is homonymous hemianopia (HH) (Zihl, 2011). In fact, HH occurs in 30% of patients that have suffered a stroke (Zhang et al., 2006a). Lesions in the primary visual cortex (V1) lead to loss of conscious access to most visual information in the contralesional visual field (Holmes, 1918; Weiskrantz et al., 1974). HH is typically defined as a visual field defect in which vision in the contralesional half of the visual field has been eliminated but no ocular damage has occurred (Holmes, 1918). It is considered to be both lateral and homonymous because it alters the same visual field expanse for both eyes. Specifically, it affects the information projected onto both the temporal hemiretina of the contralesional eye and the nasal hemiretina of the ipsilesional eye. Accordingly, a patient with a right occipital lesion will present with left HH (**Figure 1**), whereas a patient with a left occipital lesion will exhibit right HH.

In most cases, the hemianopic defect is congruent: the losses to the contralesional field of each eye are symmetric to the point that they can be superimposed (Zihl, 2011). A hemianopia that affects the vertical meridian (i.e., the entire lateral visual field) is known as a *hemianopia without macular sparing*. Contrariwise, an incomplete hemianopia, whereby the lateral visual field is partially preserved because the central portion (macula) is conserved, is known as a *hemianopia with macular sparing* (Zihl, 2011; **Figure 1B**). Macular sparing is observed when the vascular territory of the occipital pole is preserved, the spared zones of the visual field corresponding to the preserved cortical regions (Zhang et al., 2006b). In addition, some authors have proposed that macular sparing stems from the bi-hemispheric cortical representation of the central field (Brysbaert, 2004).

Homonymous hemianopia can occur before or after the onset of cortical blindness or can even occur directly after a unilateral retrochiasmatic lesion. The most common etiology of HH is stroke (whether ischemic or hemorrhagic). Indeed, 70% of strokes that involve the posterior cerebral arteries lead to HH (Pambakian and Kennard, 1997). However, HH can also arise following cerebral anoxia, an occipital lobectomy, traumatic brain injury or an arteriovenous malformation. HH can also be observed in the context of various diseases, including cancer, degenerative disorders such as posterior cortical atrophy (Benson’s syndrome) (Benson et al., 1988; Delaj et al., 2010; Zihl, 2011), or even progressive multifocal leukoencephalopathy, a condition that occurs in some HIV-positive patients (Diller and Thompson, 2007). There has been a report of a transitory case of HH during the migraine aura of a young patient (Goodwin, 2011); however, this case was highly rare. Very infrequently, HH can be an early sign of HIV infection (Gharai et al., 2012) or of epilepsy (status epilepticus amauroticus; Shaw et al., 2012). Nevertheless, HH is dictated by lesion topography and extent rather than by lesion type (Tant et al., 2002). In terms of lesion location, 40% of HH cases involve lesions of the occipital lobe; 30%, of the parietal lobe; 25%, of the temporal lobe; and 5%, of the optic tract or of the lateral geniculate nucleus (Huber, 1992). Regarding lesion side, our group recently demonstrated that the defects in hemianopic patients, as well as the cortical reorganization that follow a V1 lesion, can depend on the hemisphere in which the lesion occurs (Cavézian et al., 2010; Perez et al., 2013). These results suggest the existence of hemispheric specialization at the occipital level, which could influence the adaptive and reorganizational phenomena that follow visual-cortex lesions and visual field defects (for a discussion, see Perez et al., 2013 and Cavézian et al., 2015). This hypothesis that hemispheric specialization exists at the occipital level will be further discussed below, with regards to the nature of the ipsilesional deficit after a left vs. right occipital lesion.

## Behavioral Consequences of A Unilateral Occipital Lesion

### Contralesional Visual Field Defect

Unlike cortically blind patients, HH patients rarely exhibit anosognosia, specific memory disorder or impaired spatial orientation. However, they can eventually suffer from subtle deficits that are not mnemonic deficits *per se* but rather the direct consequence of the visual field defect (Papageorgiou et al., 2007). Indeed, as vision is cardinal for perception, attention, and memorization, visual field defects can severely impair the perception, attention, and fixation of new and relevant visual information (Kerkhoff, 2000). Furthermore, considering that vision is the most widely used sensory function for daily tasks, HH is particularly debilitating in terms of patient autonomy (de Haan et al., 2015). Indeed, many HH patients report difficulties just walking around, especially outdoors or in unfamiliar locations (Marigold et al., 2007), where they tend to bump into people or obstacles that they cannot perceive in their blind visual field. They also have trouble finding objects in their blind visual field, which considerably limits their ability to drive. HH patients also typically complain that they cannot adequately construct a full scene of their visual environment (Pambakian and Kennard, 1997). Finally, HH patients also have difficulty in reading (Leff et al., 2000; McDonald et al., 2006), notably in reading whole words, in knowing when a line of text ends (particularly in cases of right HH), and in locating the next line of text (particularly in cases of left HH) (Zihl, 2011). They can also suffer from *hemianopic alexia* (McDonald et al., 2006), which Leff et al. (2000) described as a reading impairment that is associated principally with right HH and characterized by two features: correct reading of isolated words, contrasted with an inability to generate the saccades required for reading a whole text. Interestingly, they observed that patients with a left medial occipital lesion exhibited hemianopic alexia, whereas patients whose lesion reached the fusiform gyrus suffered from pure alexia (McDonald et al., 2006). Nonetheless, in addition to the deleterious consequences of contralesional visual field defect on the ensemble of activities in which vision is crucial, it was recently discovered that HH patients can also exhibit impairments in their central visual field and in their ipsilesional visual field (Cavézian et al., 2010; Bola et al., 2013b). This phenomenon, which has been named *sightblindness* (an approximate inversion of the term *blindsight*; see below), refers to the existence of visual deficits in the visual field that is (mistakenly) considered to be unaffected (Bola et al., 2013a). We describe both *blindsight* and *sightblindness* in the following section.

### Implicit Perception in the Blind Visual Field

#### Blindsight: Definitions

Over the past four decades, researchers have found that many HH patients do not suffer from a total of loss of vision in the contralesional visual field, but rather retain certain implicit visual capabilities that enable them to unconsciously respond to stimuli presented in this (supposedly) blind field. These capacities are collectively known as *blindsight* (Poppel et al., 1973; Weiskrantz et al., 1974). Poppel et al. (1973) reported that patients who had suffered visual field defects after an occipital lesion were, upon command, able to orient a saccade toward a light stimulus positioned on their blind visual field, despite affirming that they were not aware of the stimulus and suggesting that they had merely responded by chance. Fayel et al. (2014) recently reproduced this experiment and obtained similar results (verbal detection reports were at chance level), thereby confirming that hemianopic patients, in response to stimuli that they do not consciously perceive, can indeed direct saccades in their blind visual field. However, in the Fayel study, saccades varied according to the defect. Compared to responses in the healthy group, saccades to the contralesional hemifield exhibited longer latencies and shorter amplitudes, whereas those to ipsilesional hemifield exhibited longer latencies only. We address the question of a subtle deficit in the ipsilesional visual field below.

Perenin and Jeannerod (1975) tested the ability of six hemianopic patients to distinguish and locate letters or geometric forms presented in their blind hemifield. The patients were able to accurately point toward a flash presented in their blind visual field, despite not consciously detecting the stimulus. Weiskrantz et al. (1974) described the surprising ability of a left hemianopic patient (known as D. B.) to process shapes and line orientation in the contralesional visual field. Blindsight has been defined as the “visual capacity in a field defect in the absence of acknowledged awareness” (Weiskrantz, 1986), and has also been named as residual or implicit visual capacities. The discovery of preserved visual capacities in hemianopic patients was extremely important, not only from a theoretical perspective, but also in terms of clinical practice, as it forced researchers to reconsider HH as a defect in conscious vision in the visual field contralateral to the retrochiasmatic lesion, rather than as a total loss of vision in the blind hemifield.

Over the past few decades, several studies have confirmed the presence of residual capacities in the blind field of hemianopic patients. These capacities are typically demonstrated using psychophysical methods known as forced choice methods, which usually involve motor responses in humans (Weiskrantz, 1986) or in monkeys (Stoerig and Cowey, 1997; Stoerig et al., 2002). Forced-choice methodologies have revealed that hemianopic patients can detect a visual stimulus in their blind visual field (Fendrich et al., 1992), locate such stimuli through saccades (Zihl and Von Cramon, 1980), or by pointing (Perenin and Jeannerod, 1975), detect moving stimuli (Riddoch, 1917), and distinguish among different objects (Weiskrantz et al., 1974) or facial expressions (Pegna et al., 2005). For example, when the hemianopic patient G. Y. was presented simultaneously with one stimulus in his affected visual hemifield and one stimulus in his normal hemifield, he was able to distinguish between them based on attributes such as color and movement (Ffytche et al., 1996; Morland et al., 1999). However, although G. Y.’s veridical luminance matches demonstrate that he had conscious access to luminance modulations in his blind field, he performed significantly poorer than normal at luminance discriminations and at comparing luminance between the two hemifields (Morland et al., 1999).

Another way to demonstrate the residual capacities of HH patients in their blind visual field is to study how a stimulus presented in that field affects a stimulus presented in their normal field. Such testing has been done with tasks like filling out forms (McCarthy et al., 2006). In these experiments, half of the stimulus shape is shown in the normal hemifield, and then the patient must guess the complete shape. The results of work in this area suggest that the blind hemifield remains capable of processing shapes. This was also reported by Marzi et al. (1986, 2009), using the Redundant Signal Effect (RSE) paradigm, whereby the reaction time required to detect two stimuli presented simultaneously in the two visual fields is compared to that required to detect either stimulus presented alone in each hemifield. In a first study, Marzi et al. (1986) tested a group of healthy controls and a group of 20 hemianopic patients with retrochiasmatic lesions, for spatial summation of pairs of flashes simultaneously presented either to the same hemifield or to opposite hemifields across the vertical meridian. For this task, the control subjects exhibited faster reaction times to a pair of stimuli (either in the same hemifield or in both hemifields) than to a single stimulus. In contrast, the hemianopic cohort did not show such inter-field summation, but like the control group, did exhibit summation within a single hemifield. However, one hemianopic patient with a unilateral lesion of the optic radiation exhibited a reliable overall inter-field summation: they demonstrated an RSE for pairs of visual stimuli presented across the vertical meridian, despite having seen only stimuli in the intact hemifield. This finding further corroborates the idea that the blind hemifield retains some processing capability. However, in a subsequent study, Marzi et al. (2009) showed that, when using short or long wavelength stimuli instead of achromatic stimuli, the RSE disappeared. This finding suggested that the implicit RSE found in hemianopic patients could be wavelength-dependent. Tamietto et al. (2010) described an analogous result in a patient with unilateral V1 loss: although he could not see a gray stimulus presented in his blind field, it influenced his behavioral and pupillary responses to stimuli that he consciously perceived in his intact field. In addition, this effect was accompanied by selective activations in the superior colliculus and in occipito-temporal extrastriate areas. However, consistently with the results of Marzi et al. (2009), when the patient was presented with purple stimuli—which predominantly draw on S-cones and thus, are invisible to the superior colliculus—rather than gray stimuli, the effect vanished and activations in the superior colliculus diminished significantly. Along these lines, several studies showed that the reaction time or a saccade to a stimulus presented in the normal hemifield can be modified by presentation of another (preceding or simultaneous) stimulus in the blind hemifield (Rafal et al., 1990; Cowey et al., 1998; Georgy et al., 2016). These observations have important implications for rehabilitation of visual defects and some authors have explored the possibility of training patients in order to strengthen their unconscious visual perception (Zihl, 1980). Along these lines, our group recently developed a technique for visual field restoration based on training and hyperstimulation of blindsight (Chokron et al., 2008; for a review, see Perez et al., 2014). In a rehabilitation study of nine HH patients that we trained for detection, localization and identification of stimuli in the blind visual field, we demonstrated the feasibility of objective restoration of the contralesional visual field, and showed that it can be observed by conventional automated perimetry (Chokron et al., 2008).

#### Blindsight: Description and Neuroanatomical Correlates

As mentioned in the previous section, blindsight was initially defined as visual abilities in the absence of reported visual awareness (Poppel et al., 1973; Weiskrantz et al., 1974). However, certain HH patients, despite rather extensive lesions of V1, demonstrate some residual conscious awareness in their contralesional visual field. Indeed, although they are unaware of the stimulus itself, these patients experience residual visual abilities that correlate to their residual conscious awareness of stimulus attributes such as the presence and direction of fast moving and/or high-contrast visual stimuli (Barbur et al., 1993; Zeki and Ffytche, 1998; Brogaard, 2015). The observation of such dissociation led Weiskrantz to distinguish between two types of blindsight in HH patients: *Type I*, which refers to non-conscious residual capacities in the blind visual field, and *Type II*, which refers to a form of residual awareness that positively correlates with their residual visual abilities (Weiskrantz, 1998; Weiskrantz et al., 1998). Type II blindsight, also known as *Riddoch syndrome*, was described in soldiers with striate cortex lesions that detected motion in their scotomatous fields but were otherwise unaware of visual stimuli or unable to characterize other attributes of such stimuli (Zeki and Ffytche, 1998; see for review and discussion Brogaard, 2015).

Christensen et al. (2008) showed that blindsight can be induced in healthy subjects via transcranial magnetic stimulation of the visual cortex. Similarly to HH patients after a V1 lesion, test subjects could not consciously perceive visual stimuli, yet corrected their reaching movements in response to the stimulus. In order to better characterize the subjects’ awareness of their residual capacities following the stimulation by TMS, the authors developed the Perceptual Awareness Scale (PAS), a four-level scale for testing visual awareness in blindsight. Thus, the subjects were asked to evaluate stimuli as either “clear image” (CI; “I know what was shown.”); “almost clear image” (ACI; “I think I know what was shown.”); “weak glimpse” (WG; “Something was there but I don’t know what.”); or “not seen” (NS). This scale was subsequently used by Overgaard et al. (2008) to characterize the HH patient G. R. They proposed that G. R.’s ability to discriminate amongst visual stimuli did not reflect unconscious vision, but instead, *conscious vision* that had been degraded. In fact, the use of such awareness scales could be invaluable for improving characterization of perception in the contralesional visual field of HH patients in clinical and experimental settings. Danckert and Rossetti (2005) proposed a new, three-category classification for the residual capacities of blindsight, comprising *action blindsight*, *attention blindsight* and *agnosopsia*. In action-blindsight, a motor action used to locate a target occurs in the blind visual field. In contrast, attention-blindsight, which relies on attentive processing, includes inhibition of return, orienting of attention, and detection of motion in the blind field. Accordingly, a motor action is not required to demonstrate this type. The two aforementioned types of blindsight appear to be underpinned by the same retinotectal pathway, which we discuss later on. Finally, the third category described by Danckert and Rossetti (2005), agnosopsia, is equivalent to Weiskrantz’s *Type I*. In fact, the term *agnosopsia* had previously been used by Zeki and Ffytche (1998) to characterize the non-conscious residual capacities of a patient asked to discriminate among stimuli presented in his blind visual field.

Interestingly, blindsight is not observed in all hemianopic patients. Consequently, some authors have suggested that residual visual capacities might be due to the presence of “islands” of preserved (and thus, functioning) neurons within the otherwise damaged visual cortex (Fendrich et al., 2001). Indeed, some researchers have demonstrated an intimate link between the visual-field zones in which a patient exhibited blindsight and the corresponding preserved zones in the striate cortex. For instance, Fendrich et al. (2001) considered that blindsight requires a functionally intact portion of the striate cortex. However, their hypothesis has not been universally accepted. In fact, it has been contradicted by studies in which blindsight-like phenomena have been demonstrated in patients following total loss of V1 (Perenin and Jeannerod, 1978; Georgy et al., 2016). Moreover, some studies based on functional brain imaging of hemianopic patients have failed to demonstrate any residual activity in the striate cortex (Stoerig et al., 1998). Finally, there has also been a seminal study (Ptito et al., 1996) in which residual visual capacities were observed in patients that had suffered damage to V1, but not in patients with damage to the superior colliculus. The aforementioned results suggest the existence of a secondary visual pathway responsible for non-conscious visual processing. From a functional perspective, this hypothesis stems directly from the fact that hemianopic patients can perceive the motion of a (not consciously detected) visual stimulus in their blind field. Indeed, Riddoch (1917) observed that patients with a V1 lesion were able to detect moving stimuli but could not detect static ones. This type of processing could be explained by the existence of a network of projections from the superior colliculus and the pulvinar toward the extrastriate areas (Rodman et al., 1989). Such a network would form a subcortical pathway, the retinotectal pathway (also known as the *extrageniculostriate pathway*), which would be responsible for blindsight. Based on work in monkeys and in humans (Humphrey and Weiskrantz, 1967), the structures implicated in blindsight were traced to the dorsal pathway. Accordingly, this pathway would receive afferent signals from subcortical structures such as the superior colliculus and the pulvinar. The fact that some patients can orient a motor action toward a target placed in their blind visual field despite not consciously perceiving it corroborates the existence of the aforementioned retinotectal pathway (Weiskrantz et al., 1974). Thus, Milner (1995) hypothesized that, in the absence of V1, the dorsal pathway could continue to function. According to this hypothesis, residual capacities in the impaired visual field in the absence of conscious perception would be enabled by a preserved network of cortical and subcortical areas, rather than by islands of functional neurons in V1 (Morland et al., 1999; Danckert and Goodale, 2000).

Recently, Schmid et al. (2010) proposed that the thalamic lateral geniculate nucleus (LGN) has a causal role in V1-independent processing of visual information. They tested the contribution of the LGN to visual functions of macaque monkeys with chronic V1 lesions, by comparing functional magnetic resonance imaging (fMRI) and behavioral measures before and after temporary inactivation of the LGN. Before inactivation, high-contrast stimuli presented to the lesion-affected visual field (scotoma) produced significant V1-independent fMRI activation in certain extrastriate cortical areas (V2, V3, V4, and V5/middle temporal [MT]), the fundus of the superior temporal sulcus (FST), and the lateral intraparietal area (LIP), and the animals correctly located the stimuli in a detection task. However, following inactivation of the LGN, virtually all extrastriate responses in the V1-lesioned hemispheres disappeared, indicating that the residual activation to stimuli presented in the scotoma had reached the extrastriate cortex via direct projections from the LGN. Moreover, inactivation of the LGN abolished the animals’ residual capacity to detect high contrast stimuli presented to the scotoma region of the visual field, demonstrating that the LGN might be the critical thalamic link supporting behavioral performance in blindsight.

Thus, blindsight would be underpinned by the subcortical pathways that bypass V1 and project directly into secondary visual areas such as V4 and V5 (for detection of motion), the thalamus, the brain stem, the hypothalamus, and even the amygdala (for interpretation of emotions; de Gelder et al., 1999; Danckert and Rossetti, 2005). For example, in a study of macaques, Schmid et al. (2013) demonstrated visually driven V4 neuronal responses in the absence of V1 input, which are sensitive for stimulus motion. Apart from discussing the role of V4 in motion processing, the authors proposed that it might contribute to V1-independent visual functions and may subtend blindsight. Detailed discussion of the neuroanatomic bases of blindsight has recently been enabled by brain-imaging data. For instance, Goebel et al. (2001) reported that the patient G. Y. exhibited extrastriate activation within his damaged hemisphere but without concurrent activation of V1. Morland et al. (1999) consider that recognition of colors and recognition of motion are handled by V5, without involvement of V1. Other authors have proposed that the amygdala might serve as a pathway for blindsight, as they observed use of this area in an emotional-analysis task performed by a cortically blind patient (Pegna et al., 2005). de Gelder et al. (1999) demonstrated use of the ventral pathway through evoked potentials. Thus, an alternative pathway to V1 would extend directly toward the secondary visual areas, at the level of the extrastriate cortex, passing through the superior colliculus and the pulvinar. Studies incorporating facial classification tasks have also corroborated the existence of a subcortical network underlying blindsight. Researchers have reported that faces expressing fear activate the amygdala via the superior colliculus and the pulvinar (Vuilleumier et al., 2003). Fearful faces would thus be preferentially processed based on low spatial frequency content, which would travel via the subcortical magnocellular pathway. While studying a patient that had become cortically blind following a bilateral occipital lesion, Pegna et al. (2005) found that he retained the ability to identify emotional facial expressions. Brain imaging revealed that this capacity involved activation of the right amygdala. Likewise, using fMRI, Morris et al. (2001) observed that emotional expressions could be non-consciously processed in the blind visual field of patients, and would thus activate a colliculo-thalamo-amygdala (subcortical) visual pathway (spared from the V1 lesion). More recently, Tamietto and de Gelder (2010) proposed the concept of “affective blindsight” to describe preservation of emotional processing in the blind visual field. According to them, the visual information related to recognition of emotions would be processed via the superior colliculus and the amygdala, thereby bypassing V1.

Intriguingly, Bridge et al. (2008) studied an HH patient (known as G. Y.) and healthy control subjects by diffusion-weighted imaging and identified the characteristic anatomic connections of three distinct pathways that could account for blindsight. The first pathway was observed in both hemispheres in the control subjects as well as in G. Y., whose left hemisphere was lesioned and whose right hemisphere was healthy. It bypasses V1 (Sincich et al., 2004) and links the LGN to the ipsilateral area of motion detection, V5/MT+. However, in what was perhaps the most surprising finding of the study, G. Y. also exhibited two other interhemispheric pathways, which were not observed in the control subjects. One of these two pathways comprised bundle of fibers crossing the splenium, which connects the LGN of one hemisphere to V5/MT+ of the other hemisphere. The other one linked the V5/MT+ of each hemisphere via a transcallosal connection. Interestingly, in a recent test of seventeen patients with V1 damage acquired during adulthood, Ajina et al. (2015a) found undamaged tracts between the LGN and hMT+ in the damaged hemisphere in all the patients that exhibited blindsight. This finding is consistent with their recent observation, in a separate fMRI study, of motion processing after V1 damage (Ajina et al., 2015b).

In addition to the aforementioned pathways, additional evidence to explain blindsight comes from fMRI studies that have demonstrated activation of extrastriate areas that are capable of detection, analysis, and recognition in forced-choice tasks involving non-consciously perceived stimuli in the blind visual field of hemianopic patients (Goebel et al., 2001). Current research on the neuroanatomic basis of blindsight is principally concerned with understanding the links among lesion location, lesion side, and conscious and non-conscious perception in the entire visual field in experimental or daily life settings (Cowey, 2010).

## Less-Studied Consequences of Unilateral Occipital Lesions

### Ipsilesional Deficit in Hemianopic Patients: Sightblindness

Several studies proposed that the ipsilesional visual field (IVF) might not be completely healthy in hemianopic patients. Indeed, Hess and Pointer (1989) proposed that spatial and temporal sensitivities in the IVF of hemianopic patients were lower than in control subjects. In the same vein, Clatworthy et al. (2013) showed that hemianopic patients present visual contrast sensitivity deficits in their IVF. Similarly, Rizzo and Robin (1996), and Poggel et al. (2011), suggested that hemianopic patients can exhibit lower sensitivity to signals, compromised processing of temporal information and longer reaction times in both hemifields, as compared to control participants. Regarding visual detection and analysis, Paramei and Sabel (2008) reported that these patients exhibited diminished abilities to detect fragmented targets among a noisy background in the IVF, whereas Schadow et al. (2009) found deficits in the early and late visual processing of Gestalt patterns in the IVF. More recently, Bola et al. (2013b) confirmed these findings and reported processing-speed deficits in a simple detection task in the IVF. The authors termed this phenomenon *sightblindness*, as the reverse situation of *blindsight* (Bola et al., 2013a): the former refers to visuo-attentional deficits in the IVF, whereas the latter refers to residual (although implicit) visual abilities in the contralesional visual field (CVF) that are highlighted in forced-choice tasks (e.g., Weiskrantz et al., 1974). Conversely to the case of blindsight, which has been extensively studied in hemianopia patients, vision quality in the central visual field and in the IVF of these patients has scarcely been assessed, and moreover, it has traditionally been assumed to remain intact. However, as recently proposed, neither the central visual field (Cavézian et al., 2010; Perez et al., 2013), nor the IVF of hemianopic patients (Bola et al., 2013a,b, Clatworthy et al., 2013; Cavézian et al., 2015) actually appear to be fully intact or functional. Moreover, as we recently proposed, and as discussed below, the nature of the task, the type of stimulus and the side of the occipital lesion might determine the central and ipsilesional visual deficit in hemianopic patients (Cavézian et al., 2010, 2015; Perez et al., 2013). These asymmetries confirm and extend previous findings from visual scene analyses in healthy subjects.

### Effect of Lesion Side on Sightblindness in Hemianopic Patients

In an fMRI study of the cortical networks underlying execution of a scene detection task (i.e., “Is there a scene on the screen?”) and a scene categorization task (i.e., “Is it a scene of a highway or a city?”) in healthy participants, we observed specific activation of the right inferior occipital cortex for the former task, and of the left middle occipital cortex for the latter task (Perez et al., 2013). These cerebral asymmetries confirmed previous neuroimaging studies that demonstrated that processing of global and local information at the occipital level involves hemispheric specialization (e.g., Fink et al., 1996; Chokron et al., 2000; Han et al., 2002; Iidaka et al., 2004; Peyrin et al., 2004; Musel et al., 2013; for a review, see Kauffmann et al., 2014). For instance, Peyrin et al. (2004) reported task-dependent hemispheric specialization in an fMRI study of healthy participants. When the subjects were asked to recognize scenes in which global forms remained visible but details were canceled out by low-pass filtering, the authors observed greater activation in the right occipital cortex. However, when the subjects had to recognize scenes in which local information and details remained visible but global form perception was attenuated by high-pass filtering, the authors found greater activation in the left occipital cortex. In line with such hemispheric specialization, a detection task can preferentially act as a basic visuospatial task requiring global information processing for which the right occipital cortex is specialized, whereas a categorization task (e.g., between highway and city scenes) can require a more local and detailed processing for identifying details (e.g., a car on the road) and thus, preferentially recruit the left occipital cortex.

Over the past 5 years, our group has suggested that the aforementioned functional lateralization of the occipital cortex might be important in the visual impairments of hemianopic patients. For instance, we have demonstrated that lesion side selectively affects a subject’s reaction time in detection or categorization tasks involving scenes that were either presented in their entirety, or were filtered in either the central visual field (Cavézian et al., 2010) or the ipsilesional visual field (Cavézian et al., 2015). Consistently with the left occipital specialization for scene categorization in healthy participants (Perez et al., 2013), right hemianopic patients (left-side lesion) were impaired only in the categorization task, especially with high-pass filtered scenes requiring detailed information processing. The right occipital lesion induced a higher deficit. Left hemianopic patients (right-side lesion) were impaired in both tasks (detection and categorization). We hypothesized that the right occipital lesion mainly impaired the detection processes, for which the right occipital cortex is specialized in healthy participants (Perez et al., 2013), and in turn, altered the processing of categorization.

We subsequently studied hemianopic patients by brain imaging and found that cortical reorganization for the detection task and the categorization task appeared to depend on lesion side (Perez et al., 2013). Among left hemianopic (right-side lesion) patients, activation of the visual pathways was bilateral, whereas in the right hemianopic (left-side lesion) patients, activation was essentially limited to the right side, regardless of the task instructions. These findings corroborate the idea that side of the occipital lesion is also influential in cortical reorganization. Accordingly, the left occipital lesion would lead to recruitment of the right occipital cortex, to compensate for the impaired visual processing normally performed by the left occipital lobe (e.g., categorization and detailed processing). In contrast, the right occipital lesion would lead to recruitment of a more extended, bi-hemispheric network, as a way to compensate for the greater deficits in both tasks. Nevertheless, further studies are needed to ascertain the extent to which this pattern of cortical reorganization accounts for the lesion-side-specific sightblindness.

There is another clinical aspect of hemianopia that has been relatively underrepresented in literature of the past decade: the incidence of hallucinations in HH patients, which was mentioned by the late Oliver Sacks in his recent book “Hallucinations” (Sacks, 2013). Indeed, in addition to exhibiting residual visual capacities in their blind visual field, as well as a subtle deficit in the ipsilesional visual field, hemianopic patients can also experience visual hallucinations in their contralesional visual field or even in their entire visual field, as we discuss in the following section.

### Visual Hallucinations in HH Patients

#### Nature and Frequency

Visual hallucinations are typically defined as visual perceptions that are completely removed from reality (i.e., perceptions without stimulus; Borruat, 1999). Thus, hallucinations are distinguished from *illusions*, which refer to inaccurate perceptions of real stimuli (Goebel et al., 2001). A hallucination in which the subject is cognizant of the unreality of their perception is often referred to as a *pseudo-hallucination* (Van der Zwaard and Polak, 2001).

The hallucinations experienced by hemianopic patients can be simple (e.g., points, lines, geometric shapes) or complex (e.g., objects, animals, people or animated scenes) and can involve the entire visual field or just part of it (Panayiotopoulos, 1999). These hallucinations are generally accompanied by euphoria, excitement or even negative feelings (Mosimann et al., 2008). Beyond hemianopia, visual hallucinations also occur in many psychiatric disorders such as psychoses (especially in schizophrenia and in chronic hallucinatory psychosis), in neurodegenerative disorders (e.g., Alzheimer’s and disease, Parkinson’s disease, and Lewy body dementia, in which hallucinations are frequent), during migraine auras, in certain cases of encephalitis, in epilepsy, and directly following consumption of certain substances (e.g., alcohol, narcotics or medication) in the absence of any neurological damage (Manford and Andermann, 1998). Intriguingly, complex visual hallucinations can be experienced by elderly subjects that do not exhibit any cognitive impairments but that do exhibit pre-chiasmatic (peripheral) visual impairments, as has been described for Charles Bonnet syndrome (Menon et al., 2003), which can be caused by various eye conditions. In most cases, the hallucination comprises visual perceptions of a “living” nature. They are especially clear and well-defined, and can be colored. However, patients with Charles Bonnet syndrome do not exhibit any other type of hallucination. In Charles Bonnet syndrome, as well as in hemianopic patients with a unilateral retrochiasmatic lesion, there is a disconnection between the eye and the visual cortex.

Indeed, in function of their visual field defect, HH patients can experience visual hallucinations in their blind visual field or in their entire visual field. These hallucinations vary in frequency and intensity: They can be instantaneous or enduring, and simple or complex. However, the origin of these hallucinations remains poorly understood, although they are thought to depend on lesion location (Alfaro et al., 2006). Thus, it has been reported that that the peri-lesional area of the visual cortex can generate visual hallucinations whose complexity depends on the lesion site (Kölmel, 1985): simple hallucinations apparently resulted from activation of the primary cortex, and complex hallucinations, from activation of the association cortex. Additionally, other studies have demonstrated that complex visual hallucinations can be triggered by stimulation of the temporo-occipital or the parieto-occipital lobe (Rafique et al., 2015).

#### Evaluating Visual Hallucinations in Homonymous Hemianopia Patients

Surprisingly, visual hallucinations in hemianopic patients have not been systematically researched. This might be partly due to the lack of a specific questionnaire that could enable practitioners to gather information by asking patients standardized questions. Recognizing this need, our group has developed such a questionnaire for hemianopic patients and more generally, for patients with cortical visual impairment, which we have named the *Questionnaire for Evaluating Visual Hallucinations in Homonymous Hemianopia Patients* (abbreviated as the “Q3H” questionnaire; Perez et al., 2014). For a given patient, this questionnaire enables characterization of their hallucinations (i.e., type, frequency, etc.), including determining the extent to which they are aware of the phenomenon; permits monitoring of their hallucinations over time; and enables correlation of their hallucination characteristics to their type of cortical visual impairment^1^. Importantly, neurology patients who experience visual hallucinations do not always mention their experiences on their own. Indeed, they are often reticent to discuss the subject, for fear of being labeled “crazy.” Thus, a questionnaire such the Q3H is crucial for ensuring that the issue of hallucinations in these patients is adequately and sensitively addressed. Although we designed this questionnaire chiefly to obtain a qualitative summary of hallucinatory experiences among HH patients, most of the answers can be scored quantitatively to provide data for statistical analysis. The English version of the Q3H questionnaire is shown in Annex 1, followed by a table showing representative quantitative answers. We hope that this questionnaire can help clinicians and researchers to address this overlooked aspect of hemianopia, by enabling them to better characterize these phenomena and to more clearly correlate hallucinations to their neuroanatomical and neurophysiological bases.

#### Physiopathology of Visual Hallucinations

The physiopathology of visual hallucinations is complex and might involve several mechanisms (Manford and Andermann, 1998). For instance, visual hallucinations can appear following a drop in blood flow to the occipital cortex (whether involving the primary cortex or involving reduced activity secondary to hypo-activity of afferent thalamic nuclei), or following an alteration in visual function (e.g., retinal macular degeneration). Visual hallucinations can also be due to dysfunctional synaptic signaling, as occurs with dopaminergic deficiency at D2 receptors, damage to the serotonergic system, or dysfunctional calcium membrane channels. In epilepsy, visual hallucinations can arise due to excitation of the temporal or parietal visual association cortex. Regarding HH and cortical blindness, visual hallucinations appear after a brain lesion (chiefly, occipital or temporal) and arise mostly in the patient’s blind visual field.

In hemianopic patients, visual hallucinations seem to result from compensatory hyperactivation of neighboring tissue (Braun et al., 2003; Rafique et al., 2015). Visual hallucinations can appear following excessive cortical compensation in the lateral temporal cortex, the striatum or the thalamus (Adachi et al., 2000). In fact, a hallmark of the nervous system is that when a region undergoes denervation or deafferentation, its cells become more excitable (Burke, 2002). Thus, a deafferented neuron can only survive if it receives sufficient input from alternative sources (i.e., via sprouting of adjacent axons), which in turn will lead to changes at the synaptic level. Pre-synaptically, these changes comprise increases in bouton size, in the total number of vesicles, and in the size of the release zone. While synapses are inactive, numerous receptors in the membrane of the post-synaptic neuron appear at its surface, and the post-synaptic membrane will respond more strongly to applied current. This can partly explain the greater excitability of the deafferented cortical cells. There are also major biochemical changes in the adjacent deafferented synapses, notably, an increase in the glutamatergic NMDA (*n*-methyl-D-aspartate) response and a decrease in GABA (γ-aminobutyric acid)_A_ and GABA_B_ responses. Thus, when considered together, the aforementioned changes could explain the greater excitability of deafferented neurons. According to this hypothesis, the frequency of hallucinations would correlate to the size and/or volume of the lesion. One factor that could also be determinant in the incidence, frequency and type of visual hallucinations is the side (i.e., left or right) of the occipital lesion that has caused the visual field defect, and/or the visual field (left or right) of apparition of the hallucinations. We discuss this possibility in the next section.

#### Neuroanatomic Correlations of Visual Hallucinations in HH

Over the past 5 years, our group and others have suggested that the side of the occipital lesion might be an important factor in the nature and severity of visual impairments in hemianopic patients. Similarly, Walters et al. (2006) investigated whether complex visual hallucinations caused by an occipital lesion might be linked to the lesion side and to patients’ emotional valence. In their study, they systematically searched for hallucinations in left or right brain-damaged patients and recorded the side of the hallucination, as well as its emotional valence. They then assessed the associated perceptual deficits, including loss of vision within a visual field (left or right), loss of vision within a visual quadrant, allochiria, or extinction upon presentation of concurrent bilateral stimuli to the left and the right visual field. Of the fifteen patients experiencing visual hallucinations within the left hemispace, ten had at least one visual field defect, all of which (10/10; 100%) were in the left visual field. In other words, among patients with a left visual field defect (right–side lesion), the hallucinations always occurred in the blind, contralesional visual field. In addition, all of the patients had associated negative affective valence to these events. With a total of ten patients experiencing visual hallucinations within the right hemispace, only four patients had demonstrated at least one visual field defect (tested by confrontation test only). All four of these (100%) were within the left visual field (right-side lesion), and three of them (75%) had an associated positive emotional valence. According to this study, it seems that the emotional valence of the hallucination depends on the side of its apparition rather than on the lesion side. However, all of the patients (100%) with visual field defects in this study had left visual field defects. No right visual field defects were detected across confrontational tasks for any of the patients. Thus, the fact that only patients with right-side lesions reported visual hallucinations suggests an effect of the lesion side on the occurrence of hallucinatory phenomena. Thus, we propose that the side of the occipital lesion determines the occurrence of visual hallucinations whereas the visual field of apparition influences the emotional valence, with more frequent hallucinations in the blind, contralesional visual field than in the ipsilesional visual field, as reported in the previously cited study by Walters et al. (2006) Regardless, further studies are necessary to elucidate the link between the lesion side, the visual field of apparition and the various parameters of visual hallucinations (nature, frequency, severity, similarity with mental imagery, emotional valence, etc.). Interestingly, preliminary results from one of our group’s current studies using the Q3H questionnaire suggest that the occurrence and type of visual hallucinations in hemianopic patients might also depend on the extent of the lesion.

## Conclusion and Perspectives

The sense of vision cannot be reduced to the mere capacity to detect a visual stimulus or simply to visual acuity. Research on the signs and symptoms of HH has revealed that a unilateral lesion in the visual pathways or in V1 has greater consequences than just visual field impairment. As described in this review, HH patients not only have difficulties perceiving information in their contralesional visual field, they can also present an ipsilesional visual field subtle deficit as well as experience phenomena such as hallucinations or residual vision in their blind visual field. The fact that HH patients can experience non-conscious perceptions, regardless of whether these correspond to reality, as in blindsight or hallucinations, poses numerous questions for clinicians and researchers. Above all, it underscores the paucity of knowledge on the links between perception and consciousness. Thus, HH can serve as an ideal pathologic model for the neuroanatomic bases of consciousness. From a clinical perspective, a better understanding of the reorganization and adaptation phenomena associated with specific lesions and their corresponding visual impairments will be invaluable for diagnosing, evaluating and treating patients of all ages (Pawletko et al., 2015). Accordingly, if future studies of visual perception in hemianopic patients confirm that the side of the retrochiasmatic lesion does indeed influence the observed visual impairments and the associated reorganization, then this finding would have major implications beyond research in hemispheric specialization. Specifically, it could ultimately enable lesion-side-specific treatment of HH patients, analogously to the care presently offered to patients with unilateral spatial neglect. Consistently with this premise, studies on blindsight and on sightblindness are not only yielding important theoretical findings, especially as concerns the arena of perception and consciousness, but are now influencing clinical practice (Perez and Chokron, 2014). Concretely, we recommend stimulation of patients’ residual visual capacities and training of their entire visual field (Chokron et al., 2008; Cavézian et al., 2010; 2015; Perez and Chokron, 2014).

## Author Contributions

All authors listed, have made substantial, direct and intellectual contribution to the work, and approved it for publication.

## Conflict of Interest Statement

The authors declare that the research was conducted in the absence of any commercial or financial relationships that could be construed as a potential conflict of interest.

## Footnotes

1 We recommend that the Q3H questionnaire be completed in conjunction with a neuropsychologic exam, a neurovisual exam, and a blindsight questionnaire, such that all clinical signs, symptoms and data can be correlated with the occurrence and intensity of visual hallucinations.
