# Supplementary material for: Behavioral Consequences and Cortical Reorganization in Homonymous Hemianopia
Source: Front Syst Neurosci. 2016 Jun 28;10:57. doi: 10.3389/fnsys.2016.00057 (PMC4923162; doi:10.3389/fnsys.2016.00057)
Supplement: Supplementary file 1 [file Data_Sheet_1.PDF]

Annex 1:
Questionnaire for Evaluating Visual Hallucinations in Homonymous Hemianopia Patients (Q3H Questionnaire) Chokron, Allali & Perez, Unité Vision & Cognition & Laboratoire de Psychologie de la Perception, CNRS UMR 8242, (2012).
(A preliminary version of the Q3H was published in french by Perez et al, 2014).

Questionnaire for Evaluating Visual **H**allucinations in **H**omonymous **H**emianopia Patients
(Q3H questionnaire)

Date: / /

Patient:

Neurovisual impairment:

For each question, simply check the corresponding answer. To convert these answers into quantitative data, follow the scoring instructions at the end of the questionnaire.

Description of the blind visual field

How would you describe your left/right visual field most of the time?

(Choose side according to the blind visual field of the patient.)

Incidence of hallucinations

Since your accident or since your visual difficulties began, have you been able to see anything or have you seen anything unusual in your left/right visual field? (Choose side according to the blind visual field of the patient.)

Never	
Sometimes	
Often	
Very often	

When you see things in your left/right visual field, do you feel like your vision has returned to normal? (Choose side according to the blind visual field of the patient.)

Yes	
No	

Do these things that you see appear only on your affected side or in your whole visual field?

Affected side only	
Whole visual field	

When you see these things, do you feel like they are real?

Yes	
No	

If patient answered "No" above, then ask the following:

Did you already see these things before your accident or visual difficulties began?

Yes	
No	

When you see these things, what do you think about them?

I know that they cannot be real.	
I think that they are real.	
I'm not sure if they are real.	

If the patient answered, "I know that they cannot be real" above, then ask the following:

How did you realize that what you were seeing was not real?

By comparing what I saw to what I perceived using another sense.	
By rational thinking (I understood that what I was seeing was impossible).	
Because someone who was present explained to me that what I saw was not real.	

In the event that the patient had already experienced visual hallucinations:

She/He openly talked about the experience either on their own or when asked about it.	
She/He did not openly talk about the experience.	

Continue...

Patient's reaction to the hallucinations

How do you feel about seeing these things?

It bothers me.	
I don't care either way.	
I enjoy it.	

Have you ever talked to anyone about these experiences before?

No.	
Yes, to family or friends.	
Yes, to researchers or healthcare providers (doctors, nurses, therapists, etc.)	

Note for interviewer: *If the patient answered "Yes, to family or friends" above, then ask the following question*:

How did your family or friends react?

They made fun of me.	
They were worried about me.	
They did not seem to care.	
They said that I was "crazy":	
Other (specify: ____)	

Accompanying stimuli

When you see things in your affected visual field, does this happen alone or together with other sensations?

Happens alone	
Happens together with other sensations:<br>(Specify: ____)	

Are these things that you see triggered by something in particular?

No	

Yes (specify the cause: ____)	

Memory of the hallucinations

How well do you remember these things that you have seen?

I remember them well and can clearly describe them.	
I remember them a little bit, like partly remembering a dream.	

Characteristics of the hallucinations

Please describe as clearly as possible, the things that you sometimes see in your left/right visual field? Note for interviewer: *Note the side (left/right) in which the hallucinations appear.*

When you see something in your left/right (affected) visual field, does it correspond to what you see in your unaffected visual field? Note for interviewer: *Choose side (left/right) according to the blind visual field of the patient.*

Yes	
No	
Sometimes	

Note for interviewer: *If the patient answered "No" or "Sometimes" above, then ask the following question*:

These things that you see:

Move	
Do not move	
Are shadows (like shadow puppets)	
Are colored	
Are spots of light	

These things that you see are:

Geometric shapes	
One object	
More than one object	
One person	
More than one person	

If you see objects or people, are they familiar or unfamiliar:

Familiar	
Unfamiliar	

In the scenes that you see, do the objects or people look normal and act normally:

Yes	
No (for example: shapes change, colors change, objects fly around, etc.)	

Are the scenes that you see actual scenes from your real life experiences?

Yes	
No	

Do the things or people that you see interact with you?

Yes	
No	

Similarities of the hallucinations to dreams, mental imagery or perception

On a scale of 0 to 5, where 0 is "no similarity" and 5 is "identical", how similar are these things that you see to:

A dream that you've have had	
Your mental imagery (something that you can picture in your mind if you try)	
A previously experienced perception	

Frequency of hallucinations

When did you start seeing these things?

Immediately after my accident our visual difficulties	
About 1 week afterwards	
More than 1 week afterwards	
At the beginning of my physical therapy or vision training	
Around the time that the vision in my affected visual field started to improve	

How often do you see these things?

I saw them only once.	
I saw them many times during the same period.	
I saw them many times, but in different periods.	

Note for interviewer: *If the patient has experienced the hallucinations many times, then ask the following question*:

The things that you saw each time were:

Identical	
Different	

Did you always see these things at the same time of day:

Yes (specify time:____)	
No	

Duration of the hallucinations

When you see these things, how long does the experience last?

A few seconds	
A few minutes	
Longer than 5 minutes (estimated duration: ___________________________)	
Continuously	

Scoring of answers to obtain data for statistical analysis

The answers to several of the questions can be numerically scored as to provide data for statistical analysis, using the table below. These include answers regarding the patient's

clinical parameters (age, underlying condition, and lesion side and location) and hallucination characteristics.

**Sex**	Male<br>Female	1<br>2
**Age**	Age (years)	
**Underlying condition**	Stroke<br>Tumor<br>Traumatic Brain Injury (TBI)<br>Other (infectious disease, anoxia, neurodegenerative disease)	1<br>2<br>3<br>4
**Lesion side**	Left unilateral<br>Right unilateral<br>Bilateral	1<br>2<br>3
**Lesion location**	Occipital lobe only<br>Visual pathways only<br>Occipital lobe + visual pathways<br>Occipital lobe + other cortical areas	1<br>2<br>3<br>4
**Cortical blindness**	Incomplete<br>Complete	1<br>2
**Bilateral damage**	Peripheral vision<br>Tunnel vision	1<br>2
**Homonymous hemianopia (HH)**	Incomplete<br>With macular sparing<br>Complete	1<br>2<br>3
**Quadranopsia**	Incomplete<br>With macular sparing<br>Complete	1<br>2<br>3
**Scotoma**	Central or cecocentral<br>Peripheral	1<br>2
**Visual hallucinations experienced**	Never<br>Previously, but not presently<br>Previously and presently	1<br>2<br>3
**Frequency of the hallucinations**	Never<br>Sometimes<br>Often<br>Very often	1<br>2<br>3<br>4
**Duration of the hallucinations**	A few seconds<br>A few minutes<br>Longer than 10 minutes<br>Continuously	1<br>2<br>3<br>4
**Periodicity of the hallucinations**	Several times during the same period<br>Several times over several periods	1<br>2
**Type of hallucinations (*)**	Type 1 (simple hallucinations)<br>Type 2 (complex hallucinations)	1<br>2
**Consistency of the hallucinations**	Always the same<br>Occasionally vary<br>Always different	1<br>2<br>3
**Regularity in timing of the hallucinations**	Always at the same time of day<br>At different times of day	1<br>2
**Motion of the objects or people in the hallucinations**	Static<br>Moving	1<br>2
**Coloration of the objects or people in the hallucinations**	No particular color<br>Colored	1<br>2
**Interaction of the hallucination with the patient**	No interaction<br>Interaction	1<br>2
**Similarity of hallucinations to dreams**	From 0 (no similarity) to 5 (identical)	
**Similarity of hallucinations to mental imagery**	From 0 (no similarity) to 5 (identical)	
**Similarity of hallucinations to a previously experienced perception**	From 0 (no similarity) to 5 (identical)	
**Hemifield involved**	Ipsilesional	1

	Contralesional<br>Bilateral	2<br>3
**Emotional response to the hallucinations**	Neutral<br>Positive or pleasant<br>Negative or troubling	1<br>2<br>3
**Critical assessment of the hallucinations**	Thought it was real<br>Was not sure<br>Knew that it was not real	1<br>2<br>3
